# Presence of mitral stenosis is a risk factor of new development of acute decompensated heart failure early after transcatheter aortic valve implantation

**DOI:** 10.1136/openhrt-2020-001348

**Published:** 2020-10-05

**Authors:** Tsukasa Okai, Kazuki Mizutani, Masahiko Hara, Tomohiro Yamaguchi, Mana Ogawa, Asahiro Ito, Shinichi Iwata, Yasuhiro Izumiya, Yosuke Takahashi, Toshihiko Shibata, Minoru Yoshiyama

**Affiliations:** 1 Cardiovascular Medicine, Osaka City University, Osaka, Japan; 2 Cardiology, Kindai University Faculty of Medicine, Osakasayama, Japan; 3 Department of Cardiovascular Medicine, Shimane University Faculty of Medicine Graduate School of Medicine, Izumo, Japan; 4 Cardiovascular Surgery, Osaka City University, Osaka, Japan

**Keywords:** aortic valve disease, heart failure, mitral stenosis

## Abstract

**Aims:**

Acute decompensated heart failure (ADHF) can occur early after transcatheter aortic valve implantation (TAVI), but the risk factors or mechanisms associated with it have not been fully determined. This hypothesis-generating study aimed to investigate the clinical indices associated with the development of ADHF within 72 hours after TAVI and to improve procedural approaches for TAVI.

**Method and results:**

In this single-centre hypothesis generating prospective observational study, we enrolled 156 consecutive patients with severe aortic stenosis who underwent TAVI between January 2016 and February 2018 at our institution. We set the primary endpoint as the new development of ADHF within 72 hours after TAVI, and clinical indices associated with it were evaluated using a multivariable logistic model. The median age of the patients was 83 (quartile range 80–86) years, 48 (30.8%) were men and the median Society of Thoracic Surgery-Predicted Risk of Mortality was 7.1 (range 5.2–10.4). Mitral stenosis (MS), defined as mean transmitral valve pressure gradient ≥5 mm Hg, was present in 15 (9.6%) patients. After TAVI, the invasive mean transaortic valve pressure gradient (mAVPG) decreased from 48 (36–66) to 7 (5–11) mm Hg, and 12 (7.7%) patients developed ADHF within 72 hours after TAVI. Multivariable logistic regression analysis showed that MS (adjusted OR, 14.227; 95% CI 2.654 to 86.698; p=0.002) and greater decreases in mAVPG (1.038; 1.003 to 1.080; p=0.044) were associated with ADHF.

**Conclusions:**

MS and drastic improvement of mAVPG were associated with new development of ADHF within 72 hours after TAVI.

**Key questions**

**What is already known about this subject?**

- Patients with mitral stenosis (MS) who underwent transcatheter aortic valve implantation (TAVI) have increased in-hospital death and 1-year mortality.

**What does this study add?**

- Patients with MS have increased risk of acute decompensated heart failure (ADHF) early after TAVI.

**How might this impact on clinical practice?**

- We should take careful management for patients with MS to avoid the development of ADHF early after TAVI.

## Introduction

Aortic stenosis (AS) causes left ventricular outflow impairment, and the subsequent pressure overload with or without left ventricular systolic dysfunction can lead to heart failure (HF).^1^ Surgical aortic valve replacement has been the mainstay of radical treatment in symptomatic patients with AS for decades.^1 2^ On the contrary, transcatheter aortic valve implantation (TAVI) has been recognised as a valid therapeutic option for patients with high surgical risk, and the non-inferior 5-year clinical outcomes compared with surgical replacement resulted in expanding its indications for patients at low risk.^3–5^ As the procedures of TAVI became less invasive with advancements of medical devices, the 30-day complication rate including life-threatening bleeding of TAVI is lower than that of surgery at present and the patients can be discharged much earlier after TAVI than after surgery.^5–7^ However, there is still an outstanding problem regarding the development of acute decompensated HF (ADHF) which occurs immediately after TAVI.^3–5 8^ Although there are a plenty of evidence with respect to HF rehospitalisation after discharge in patients who underwent TAVI, few clinical studies have focused on ADHF in the early phase after TAVI and the risk factors or mechanisms associated with it had not been fully determined.^3–5 8–14^ Based on these perspectives, the purpose of this hypothesis-generating study was to investigate the clinical indices associated with the development of ADHF within 72 hours after TAVI and to identify procedural approaches for better TAVI by managing remaining risk of ADHF which occurs in the early phase after TAVI.

## Methods

### Study population

**Figure 1:**
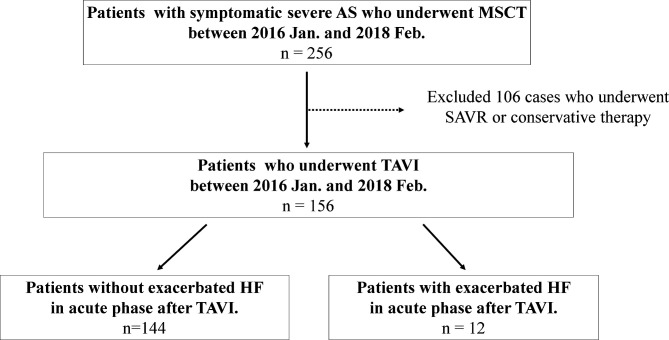
Flow chart of patient selection. AS, aortic stenosis; HF, heart failure; MSCT, multislice CT; SAVR, surgical aortic valve replacement; TAVI, transcatheter aortic valve implantation.

This single-centre prospective observational study included 156 consecutive patients with symptomatic severe AS who underwent TAVI at Osaka City University Hospital between January 2016 and February 2018 (figure 1). TAVI at our institution during the study period was indicated for patients at high risk for surgery. The inclusion criteria were as follows: (1) presence of symptoms, (2) presence of degenerative AS, (3) an estimated mean transaortic valve pressure gradient (mAVPG) of >40 mm Hg or a jet velocity of >4.0 m/s, and/or (4) an aortic valve area <1.0 cm^2^ (or an effective orifice area index <0.6 cm^2^/m^2^) by transthoracic echocardiography (TTE), according to the guideline for valvular heart disease of the European Society of Cardiology and the European Association for Cardio-Thoracic Surgery.^15^ The indication and surgical risk for TAVI were determined based on the clinical consensus of a heart team comprised of cardiac surgeons, interventional cardiologists, anaesthesiologists and imaging specialists. For example, patients with a Society of Thoracic Surgery-Predicted Risk of Mortality (STS-PROM) score≧8, patients with frailty, and patients aged≧80 years were considered to be high surgical risk in the present study. Written informed consent was obtained from all patients. The authors had full access to the data and were responsible for its integrity. All authors have read and agreed to the manuscript as written.

### Standard procedure of TAVI

We chose the transfemoral approach as the first option when patients did not have an excessively narrow access route for insertion of the sheath or aortic arch atheroma. We performed TAVI under general anaesthesia in a hybrid operating room, except for two patients who underwent conscious sedation due to pulmonary dysfunction. Transcatheter heart valves were classified as balloon-expandable (Edwards Sapien XT or Sapien 3 Transcatheter Heart Valve; Edwards Lifesciences, Irvine, California, USA) or self-expandable (Medtronic classic CoreValve or CoreValve EvolutR; Medtronic, Minneapolis, Minnesota, USA). Balloon-expandable valves were the first choice, and self-expandable valves were reserved for patients with a narrow aortic annulus. We performed simultaneous catheter measurement to evaluate the invasive mAVPG as well as a measurement of left ventricular end-diastolic pressure before and after TAVI. After successful TAVI, patients were given a saline solution by intravenous infusion (1 mL/kg/hour) until the start of ingestion. The brachial blood pressure was maintained at less than 130 mm Hg using calcium channel blocker, if necessary.

### Data collection

All data shown in the tables and figures were collected prospectively from patient records. Because this study aimed to generate hypothesis, preprocedural TTE evaluated AS-related indices as well as mitral stenosis (MS)-related findings such as the transmitral valve pressure gradient and presence or absence of mitral annular calcification (MAC). We defined significant MS as a mean mitral pressure gradient ≧5 mm Hg and defined MAC as a structure producing intense echocardiographic signals located at the junction of the atrioventricular groove and posterior mitral leaflet, although severe AS could result in low-flow, low-gradient MS with prolonged pressure half time which is related to impaired left ventricular relaxation.^16–18^ We also assessed the left ventricular diastolic function using transmitral E/A value with pulse-wave Doppler or the E/e’ value measured at the mitral annulus septum with tissue Doppler for reference. However, it is difficult to evaluate diastolic function with E/e' in the presence of MS, as stated in the American Society of Echocardiography guidelines.^19 20^ Multislice CT data were measured with the SYNAPSE VINCENT (Fujifilm, Japan). We defined areas with CT value of ≧600 Hounsfield units as calcification and individually measured the calcium volume of the aortic valve complex and that of the mitral valve apparatus (leaflet and annulus).^21^

### Endpoint and statistical analysis

We set the primary endpoint as the new development of ADHF within 72 hours after the procedure and the objective of the present study was to investigate its risk factors. We defined the new development of ADHF after TAVI as worsening of HF symptoms, such as dyspnoea with objective signs of congestion like pulmonary oedema or hypoperfusion requiring treatments such as intravenous administration of diuretics, vasodilator, inotropes or respiratory supports based on recommendations by international guidelines.^22^

Continuous variables were summarised using medians and IQR (quartiles 1–3), and categorical variables were summarised using means of counts and percentages. We first divided our patients into two groups depending on the presence or absence of ADHF within 72 hours after TAVI and compared patient backgrounds because this is the hypothesis-generating study. Differences in continuous and categorical variables between groups were compared using the Wilcoxon rank-sum test and the χ^2^ test, respectively. Then, the prespecified analysis included the evaluation of the risk factors of the new development of AHDF in the early phase after TAVI using univariable logistic regression analysis with its 95% CI. Since the absolute number of primary endpoint was estimated to be low, ad hoc adjustments of confounding factors by multivariable regression model was admitted only as a reference in the prespecified statistical protocol. In the multivariable model, we determined the confounders statistically based on the Akaike information criteria (AIC) and also performed conventional multivariable model employing variables, which showed statistical significance in the univariable logistic regression model. To avoid the problem of multicollinearity, New York Heart Association (NYHA) functional classification and urgency of the procedure were not used because these indices are included in the STS-PROM score, and invasive mAVPG improvement was selected as a representative value for preprocedural and postprocedural severity of AS in a multivariable model. In addition, we employed classification and regression tree (CART) analysis to reveal the most important risks among these variables.^23^ Furthermore, since AIC-based multivariable logistic regression and CART analyses revealed that the presence of MS was strongly associated with primary endpoint, we estimated and compared 2-year survival with its 95% CI after TAVI using the Kaplan-Meier method in patients with or without MS. The difference between groups was evaluated using the log-rank test. Statistical analyses were performed using R software packages (V.3.3; R Development Core Team). The significance level of a statistical hypothesis testing was set at 0.05 and the alternative hypothesis was two sided.

## Results

Patient characteristics are shown in table 1. ADHF occurred in 12 patients at a median interval of 20 hours (IQR 18–23) after TAVI. In the total study population, the median patient age was 83 (80–86) years, and 30.8% were male. The median STS-PROM score, brain natriuretic peptide (BNP) level on admission and estimated mAVPG on TTE were 7.1% (5.2–10.4), 200 pg/mL (80–405) and 49 mm Hg (37–64), respectively. Significant MS was present in 9.6% of the patients, and MAC was detected in 63.5%. The E/e’ value was 23.8 (19.1–31.2). No significant differences in patient characteristics between groups with or without the development of ADHF, except for the presence of NYHA HF functional class III or IV, STS-PROM score, BNP level on admission and prevalence of significant MS.

**Table 1:** Patient characteristics

Parameter	Total (n=156)	Heart failure (−) (n=144)	Heart failure (+) (n=12)	P value
Patient characteristics				
Age (years)	83 (80–86)	83 (80–86)	83 (83–86)	0.439
Male sex, n (%)	48 (30.8)	45 (31.2)	3 (25.0)	0.652
BSA (m^2^)	1.41 (1.29–1.54)	1.41 (1.31–1.54)	1.29 (1.23–1.47)	0.070
Coronary risk factors and medical history				
Hypertension	150 (96.2)	139 (96.5)	11 (91.7)	0.400
Dyslipidaemia	86 (55.1)	78 (54.2)	8 (66.7)	0.403
Diabetes mellitus	33 (21.1)	32 (22.2)	1 (8.3)	0.258
Current smoking	11 (7.1)	10 (6.9)	1 (8.3)	0.857
Coronary artery disease	43 (27.6)	40 (27.8)	3 (25.0)	0.836
Atrial fibrillation	31 (19.9)	29 (20.1)	2 (16.7)	0.772
Previous CABG	4 (2.6)	4 (2.8)	0 (0.0)	0.559
Previous myocardial infarction	9 (5.8)	9 (6.2)	0 (0.0)	0.372
Previous PCI	28 (18.0)	26 (18.1)	2 (16.7)	0.904
Previous stroke	19 (12.2)	17 (11.8)	2 (16.7)	0.621
Pulmonary disease	30 (19.2)	29 (20.1)	1 (8.3)	0.319
Liver disease	7 (4.5)	7 (4.9)	0 (0.0)	0.435
NYHA Class III or Ⅳ	62 (39.7)	54 (37.5)	8 (66.7)	0.047
Clinical Frailty Scale	4 (3–4)	4 (3–4)	4 (3–4)	0.724
STS-PROM score	7.1 (5.2–10.4)	7.0 (5.0–9.9)	10.4 (7.4–13.1)	0.006
Laboratory data on admission				
Haemoglobin (g/L)	1.15 (1.03–1.27)	1.16 (1.03–1.27)	1.09 (1.06–1.18)	0.431
e-GFR (mL/min/1.73 m2)	49.9 (40.3–61.1)	50.2 (40.5–62.2)	41.5 (34.0–51.6)	0.113
Albumin (g/dL)	3.8 (3.5–4.1)	3.8 (3.5–4.1)	3.6 (3.4–3.8)	0.229
Na (mEq/L)	140 (139–142)	140 (139–142)	141 (139–142)	0.933
BNP (pg/mL)	200 (80–405)	187 (76–386)	600 (254–1286)	0.006
Drugs				
ACE-I or ARB	95 (60.9)	89 (61.8)	6 (50.0)	0.421
β blocker	44 (28.2)	39 (27.1)	5 (41.7)	0.281
Ca blocker	78 (50.0)	71 (49.3)	7 (58.3)	0.548
Diuretic	83 (53.6)	74 (51.7)	9 (75.0)	0.121
Tolvaptan	23 (14.7)	20 (13.9)	3 (25.0)	0.297
Statin	64 (41.0)	59 (41.0)	5 (41.7)	0.963
TTE data on admission				
LVEF (%)	60 (55–65)	60 (55–65)	60 (53–64)	0.754
LV diastolic diameter (mm)	43 (39–46)	43 (39–46)	42 (39–44)	0.385
LV systolic diameter (mm)	25 (21–29)	25 (21–30)	25 (20–27)	0.670
Left atrial diameter (mm)	43 (39–46)	43 (38–46)	45 (43–47)	0.154
Mean AVPG (mm Hg)	49 (37–64)	49 (37–61)	55 (47–76)	0.231
Peak AVPG (mm Hg)	84 (68–108)	83 (68–106)	94 (80–121)	0.317
AVA index (cm^2^/m^2^)	0.45 (0.40–0.52)	0.45 (0.40–0.53)	0.44 (0.43–0.49)	0.620
Moderate or severe AR	22 (14.1)	21 (14.6)	1 (8.3)	0.550
Moderate or severe MR	22 (14.1)	19 (13.2)	3 (25.0)	0.259
Moderate or severe TR	13 (8.3)	11 (7.6)	2 (16.7)	0.277
Significant MS	15 (9.6)	10 (6.9)	5 (41.7)	<0.001
MAC	99 (63.5)	92 (63.9)	7 (58.3)	0.701
E/A	0.69 (0.55–0.83)	0.67 (0.55–0.81)	0.83 (0.70–0.92)	0.174
E/e’	23.8 (19.1–31.2)	23.6 (18.8–31.2)	29.2 (22.5–36.7)	0.079
Preprocedural CT data				
Annular area (mm^2^)	385 (342–442)	388 (342–447)	368 (348–386)	0.338
Perimeter (mm)	69.7 (65.6–74.6)	69.9 (65.6–74.7)	68.1 (66.2–70.5)	0.376
Calcium volume of AV (mm^3^)	488 (336–722)	490 (340–735)	384 (308–605)	0.497
Calcium volume of MV (mm^3^)	41 (0–472)	41 (0–472)	108 (0–478)	0.959

Categorical variables are shown as numbers (percentages) and continuous variables are shown as medians (25–75th percentiles).

ACE-I, ACE-inhibitor; AR, aortic regurgitation; ARB, angiotensin II receptor blocker; AV, aortic valve; AVA, aortic valve area; AVPG, aortic valve pressure gradient; BNP, brain natriuretic peptide; BSA, body surface area; CABG, coronary artery bypass graft; EF, ejection fraction by modified Simpson methods; e-GFR, estimated glomerular filtration rate; LV, left ventricle; MAC, mitral annular calcification; MR, mitral regurgitation; MS, mitral stenosis; MV, mitral valve; NYHA, New York Heart Association; PCI, percutaneous coronary intervention; STS-PROM, Society of Thoracic Surgery-Predicted Risk of Mortality; TR, tricuspid regurgitation; and TTE, transthoracic echocardiography.

**Figure 2:**
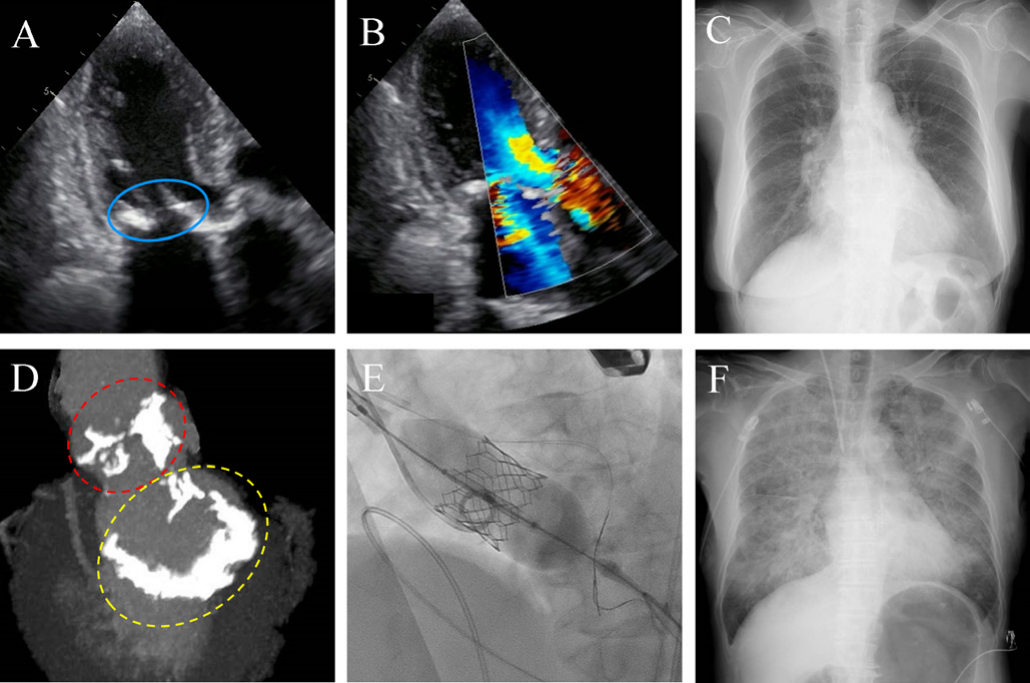
A representative case. (A): Preprocedural TTE image (parasternal long-axis view) LVEF was 63%, mean AVPG was 49 mm Hg, and AVAi was 0.41 cm^2^. The light blue circle indicates MAC. (B): Preprocedural TTE image (parasternal short-axis view). Restricted opening due to MAC. The mean AVPG was 15 mm Hg. The light blue circle indicates MAC. (C): Chest X-ray image before TAVID: Preprocedural MSCT findings. The red-dot circle indicates the aortic valve complex calcium with volume of 259 mm^3^. The yellow circle indicates the mitral valve calcification with a volume of 1923 mm^3^. (E): Transfemoral TAVI Implantation of a 23 mm Sapien three decreased the mean AVPG from 63 mm Hg to 9 mm Hg. (F): Chest X-ray image 2 hours after TAVI. The patient had shortness of breath and was diagnosed with development of ADHF. AVAi, aortic valve area index; ADHF, acute decompensated heart failure; AVPG, aortic valve pressure gradient; LVEF, left ventricular ejection fraction; MAC, mitral annular calcification; MSCT; multislice CT; TAVI, transcatheter aortic valve implantation; TTE, transthoracic echocardiography.

Table 2 and online supplemental table 1 show the procedural and outcome information. In the total study population, 85.3% of the patients underwent transfemoral TAVI, and 89.1% underwent balloon-expandable TAVI. Invasive mAVPG decreased from 48 (36–66) mm Hg to 7 (5–11) mm Hg, with the simultaneous mAVPG improvement of 40 (30–59). This mAVPG improvement as well as the incidence of urgent TAVI procedure and mAVPG before TAVI were the only indices that showed statistically significant differences between groups in the table 2. Regarding the in-hospital prognosis, two patients died in hospital due to lethal retroperitoneal haemorrhage and left main trunk occlusion 3 days and 31 days after TAVI, respectively. Otherwise, 1.3% of the patients suffered from disabling stroke, 3.2% from coronary occlusion and 5.1% from acute kidney injury and 4.5% needed permanent pacemaker implantation. A representative case of a new development of ADHF after successful TAVI is shown in figure 2.

10.1136/openhrt-2020-001348.supp1 Supplementary data

**Table 2:** Procedural and outcome information

Parameter	Total (n=156)	Heart failure (−) (n=144)	Heart failure (+) (n=12)	P value
Procedural data				
Access route				0.067
Transfemoral	133 (85.3)	125 (86.8)	8 (66.7)	
Transapical	20 (12.8)	17 (11.8)	3 (25.0)	
Transsubcravian	1 (0.6)	1 (0.7)	0 (0.0)	
Direct-Aorta	2 (1.3)	1 (0.7)	1 (8.3)	
Valve type				0.753
Edwards SAPIEN XT	46 (29.5)	43 (29.9)	3 (25.0)	
Edwards SAPIEN 3	93 (59.6)	86 (59.7)	7 (58.3)	
Medtronic corevalve	3 (1.9)	3 (2.1)	0 (0.0)	
Medtronic EvolutR	14 (9.0)	12 (8.3)	2 (16.7)	
Valve size (mm)	23 (23–26)	23 (23–26)	23 (23–26)	0.604
Urgency	20 (12.8)	16 (11.1)	4 (33.3)	0.027
Simultaneous catheter measurement				
Mean AVPG before TAVI	48 (36–66)	48 (35–66)	65 (57–96)	0.034
Mean AVPG after TAVI	7 (5–11)	7 (5–11)	8 (6–9)	0.795
Mean AVPG improvement	40 (30–59)	39 (29–57)	60 (49–85)	0.023
LVEDP before TAVI	17 (14–23)	17 (14–23)	21 (15–24)	0.474
LVEDP after TAVI	20 (13–25)	20 (14–25)	16 (10–24)	0.529
PVL grade after TAVI				0.162
None	26 (18.6)	26 (20.0)	0 (0.0)	
Trace	79 (56.4)	70 (53.8)	9 (90.0)	
Mild	34 (24.3)	33 (25.4)	1 (10.0)	
Moderate	1 (0.7)	1 (0.8)	0 (0.0)	
Severe	0 (0.0)	0 (0.0)	0 (0.0)	
Contrast (mL)	66 (57–84)	66 (56–84)	66 (59–74)	0.878
Fluoroscopy time (min)	19 (13–29)	19 (13–29)	21 (12–25)	0.939
Procedure time (min)	72 (50–102)	71 (50–101)	93 (51–129)	0.333
Anaesthesia time (min)	132 (109–165)	132 (109–162)	153 (119–188)	0.212
Volume of infusion (mL)	1300 (838–1623)	1290 (800–1603)	1595 (925–2490)	0.204
Blood transfusion (mL)	0 (0–280)	0 (0–280)	140 (0–615)	0.073
In-out balance (mL)	950 (565–1405)	940 (550–1355)	1395 (785–1940)	0.107
Periprocedural complications				
In-hospital death	2 (1.3)	2 (1.4)	0 (0.0)	0.681
Disabling stroke	2 (1.3)	2 (1.4)	0 (0.0)	0.681
Coronary occlusion	5 (3.2)	5 (3.5)	0 (0.0)	0.512
Acute kidney injury	8 (5.1)	7 (4.9)	1 (8.3)	0.600
Permanent pacemaker implantation	7 (4.5)	6 (4.2)	1 (8.3)	0.503

Caption is the same as in table 1.

AVPG, aortic valve pressure gradient; LVEDP, left ventricular end diastolic pressure; PVL, paravalvular leakage; TAVI, transcatheter aortic valve implantation.

**Figure 3:**
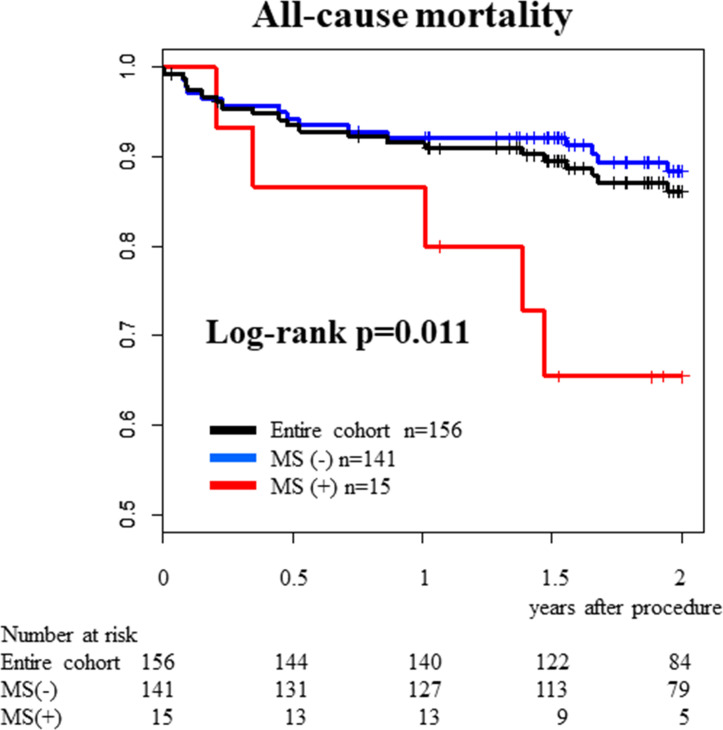
Kaplan-Meier survival estimates. MS, mitral stenosis.

Table 3 shows the results of prespecified univariable and ad hoc multivariable logistic regression analyses. In the univariable analysis, STS-PROM score (unadjusted OR (OR) 1.159; 95% CI 1.049 to 1.287; p=0.004), BNP levels on admission (OR 1.014; 95% CI 1.004 to 1.024; p=0.004 per 10 pg/mL increase), MS (OR 9.571; 95% CI 2.466 to 35.977; p<0.001) and invasive mAVPG improvement after TAVI (OR 1.048; 95% CI 1.015 to 1.086; p=0.006) were significantly associated with the new development of ADHF within 72 hours after TAVI. AIC-based variable selection left MS and mAVPG improvement as final covariates for best predictive model, and the multivariable analysis showed that MS and a greater decrease of mAVPG after TAVI had a statistically significant effect on the primary endpoint with adjusted OR of 14.227 (95% CI 2.654 to 86.698; p=0.002) and 1.038 (95% CI 1.003 to 1.080; p=0.044). These results are consistent with those of the conventional multivariable model. In addition, the CART analysis suggested that the presence of MS is the strongest risk factor for developing ADHF. The estimated 2-year mortality rate was 34.5% (95% CI 16.0% to 64.3%) for the MS group vs 11.6% (95% CI 7.1% to 18.6%) for the no MS group (log-rank p=0.011) (figure 3).

**Table 3:** Associations between each variable and the development of acute decompensated heart failure after TAVI

Parameter	Univariate		AIC-based multivariable model		Conventional multivariable model	
	Unadjusted OR (95% CI)	P value	Adjusted OR (95% CI)	P value	Adjusted OR (95% CI)	P value
STS-PROM score	1.159 (1.049 to 1.287)	0.004	–	–	1.112 (0.920 to 1.318)	0.233
BNP (per 10 pg/mL increase)	1.014 (1.004 to 1.024)	0.004	–	–	0.988 (0.963 to 1.011)	0.333
Significant MS	9.571 (2.466 to 35.977)	<0.001	14.227 (2.654 to 86.698)	0.002	16.798 (2.651 to 129.450)	0.003
Mean AVPG improvement (catheter measurement)	1.048 (1.015 to 1.086)	0.006	1.038 (1.003 to 1.080)	0.044	1.050 (1.004 to 1.108)	0.0499

Caption is the same as in table 1.

AIC, Akaike information criteria; AVPG, aortic valve pressure gradient; BNP, brain natriuretic peptide; MS, mitral stenosis; STS-PROM, Society of Thoracic Surgery-Predicted Risk of Mortality; TAVI, transcatheter aortic valve implantation.

## Discussion

In this hypothesis-generating single-centre prospective observational study, the incidence of ADHF after TAVI was 7.7%. In addition, both univariable and multivariable logistic regression analyses revealed that the presence of significant MS (mean pressure gradient ≧5 mm Hg) and improvement of mAVPG were associated with new development of ADHF within 72 hours after TAVI, with the adjusted OR of MS much higher than that of mAVPG which also indicated its importance in the CART analysis. Since few studies have focused on the epidemiology and risk factors of the new development of ADHF within 72 hours after TAVI, our study could provide physicians with new insights into the management strategies of TAVI in patients with severe AS complicated with MS.^24^

### Mechanism of developing ADHF after TAVI

In general, the pathophysiology of developing HF is considered multifactorial, and numerous disparate aetiologies are indicated in previous reports such as left ventricular dysfunction, coronary artery diseases and valvular diseases.^22^ Furthermore, alternative access, such as transapical TAVI, could lead to the development of HF due to its invasiveness compared with the transfemoral approach.^25^ However, the risk factors or mechanisms of ADHF early after TAVI has yet to be fully elucidated although in-hospital ADHF after TAVI is sometimes encountered in the clinical setting.^3–5 8–14^ Based on the results of the present study, we hypothesised that rapid worsening of haemodynamics associated with increased cardiac output after TAVI and resultant increase of left atrial pressure due to severe MS play an important role in the development of ADHF early after TAVI. That is, in the first step, TAVI could immediately release the left ventricular outflow obstruction, which then leads to a drastic increase in cardiac output.^26^ Actually, it is reported that cardiac index increase from 2.0±0.6 L/min/m^2^ to 3.1±0.7 after TAVI.^26^ We also hypothesised that a greater decrease in mAVPG, namely greater improvement of mAVPG through greater release of the left ventricular outflow obstruction, can be associated with higher cardiac output and resulting high-output HF. However, in the next step, the left atria with significant MS could not sufficiently handle the increased left atrial volume overload from the pulmonary vein. This also led to severe left atrial volume overload, increased the left atrial and pulmonary artery wedge pressures, and led to rapid progression of pulmonary oedema. Considering these, it is intuitively understandable that both MS and improvement of mAVPG were associated with the new development of ADHF early after TAVI in the present study. Thus, we hypothesised that ADHF after TAVI is categorised as so-called high-output HF, which easily emerges when complicated with MS.

### Clinical implication

We believe that our results remind us one important and two possible clinical implications. First, it is noteworthy that the OR of significant MS was much greater than those of other variables and that CART analysis suggested that the most predictive index of new development of ADHF early after TAVI was significant MS. Since the high incidence of comorbid MS (>10%) due to degenerative MAC (>45%) in patients who undergo TAVI, it is important to know the potential risk of MS.^13 27 28^ Preprocedural risk stratification and risk assessment such as in-hospital ADHF early after TAVI may contribute to the improvements of outcomes in AS patients who undergo TAVI. Actually, Joseph *et al* reported that severe MS was an independent predictor of in-hospital death and indicated that severe MS was an independent predictor of 1-year mortality and HF-related hospitalisation.^13^ Furthermore, Asami *et al* reported that patients with MS had an increased risk of cardiovascular death both at 30 days (adjusted HR 4.05: 95% CI 2.10 to 7.82) and 1 year (adjusted HR 3.64: 95% CI 2.38 to 5.56).^29^ Considering the above-mentioned possible mechanisms of developing ADHF early after TAVI, it is intuitively understandable that MS may affect not only the development of in-hospital ADHF but also long-term prognosis after TAVI in the same mechanisms through high-output type haemodynamic impairment. Actually, this is consistent with our results demonstrating higher 2-year mortality in patients with significant MS than those without MS (figure 3). Second, we speculated that measurements of cardiac output, transmitral valve pressure gradient, left atrial pressure using pulmonary artery wedge pressure, especially after TAVI, may be recommended in assessing the risk of ADHF when treating patients with severe AS complicated with significant MS. Finally, risks and benefits of concomitant management of significant MS can be next objectives when external validity of the risk of significant MS after TAVI was established although definite management choices are unknown for the management of significant MS at present.

### Study limitations

This study has several limitations due to the nature of single-centre design. First, the small study population (n=156) and low incidence of primary endpoint underpowered the statistical analysis, and there are some differences regarding baseline characteristics, such as AS severity and HF condition, between study groups even though we tried to minimise these differences using multivariable models. The management of perioperative HF in the ADHF group was insufficient partly because we could not help but manage AS patients complicated with MS in a wet volume condition, in order to avoid low-output HF associated with AS and MS. Second, although we defined significant MS as a mean mitral pressure gradient ≧5 mm Hg according to the guidelines of the American and European Society of Echocardiography, severe AS could underestimate the mitral valve pressure gradient.^18^

Also, the planimetry data using three-dimensional transoesophageal echocardiography were not available although it is prioritised for the diagnosis of MS complicated with AS in the current guidelines, because we were unaware of the guidelines when we planned the study in 2016.^18^ Third, external validity cannot be secured and should be evaluated in future studies. Lastly, we did not perform direct measurements of cardiac output and pulmonary artery wedge pressure, although these two factors may be important to verify our above-mentioned hypothesis that rapid worsening of haemodynamics is associated with increased cardiac output after TAVI and resultant increase of left atrial pressure due to severe MS. Hence, readers should keep in mind these limitations when interpreting these results, especially considering the critical limitation of low incidence of the primary endpoint, although that we employed two kinds of analyses in order to validate a robustness of our results.

In conclusion, MS and drastic improvement of mAVPG were associated with the new development of ADHF within 72 hours after TAVI.

In-out balance was calculated as a volume of infusion and blood transfusion minus urine output.
