# Supplementary material for: Presence of mitral stenosis is a risk factor of new development of acute decompensated heart failure early after transcatheter aortic valve implantation
Source: Open Heart. 2020 Oct 5;7(2):e001348. doi: 10.1136/openhrt-2020-001348 (PMC7537436; doi:10.1136/openhrt-2020-001348)
Supplement: Supplementary data [file openhrt-2020-001348supp001.pdf]

Supplemental Table 1. Degree of Mitral Valve Pressure Gradient after Transcatheter Aortic Valve Implantation in Patients with Significant Mitral Stenosis

Parameter	Heart Failure (-) (n=10)	Heart Failure (+) (n=5)	p-value
Mean MVPG (mmHg)	6.0 (5.8-7.0)	7.0 (7.0-8.0)	0.132

MVPG, mitral valve pressure gradient
